# Advanced viral genome *in vitro* Cas9 editing (AdVICE): an overnight method for traceless and limitless manipulation of adenoviral and vector genomes with large transgenes

**DOI:** 10.1128/jvi.02265-24

**Published:** 2025-05-21

**Authors:** Jean-Baptiste Vergnes, Benoit Roger, Richard Iggo, Harald Wodrich

**Affiliations:** 1 Microbiologie Fondamentale et Pathogénicité, MFP CNRS UMR5234, University of Bordeaux, Bordeaux, France; 2 INSERM U1312, University of Bordeaux, Bordeaux, France; International Centre for Genetic Engineering and Biotechnology, Trieste, Italy

**Keywords:** adenovirus, recombineering, vectors, CRISPR, gibson assembly

## Abstract

The size and complexity of large viral genomes limit the technical possibilities for genome manipulations in fundamental research and medical or technological applications. State-of-the-art recombineering in bacteria has partially overcome this limit but remains a time-consuming and complex procedure requiring specialist expertise. Here, we describe a simplified and highly efficient *in vitro* protocol for unlimited and traceless manipulation applicable to large viral genomes from DNA viruses using a combination of CRISPR/Cas9 cleavage and *in vitro* DNA assembly. We successfully used the protocol to manipulate adenovirus genomes, showing that genome rescue from viruses, insertions, deletions, and mutagenesis can be performed in a simple overnight procedure in a standard laboratory setting without the need for advanced knowledge of molecular biology. Finally, we use our approach to demonstrate the *de novo*, multi-step construction of an adenovirus vector suitable for delivering very large transgenes for gene editing.

**IMPORTANCE:**

The 36 kb size of the adenoviral genome has long been a deterrent to the construction of adenoviral mutants by scientists wishing to study the virus itself or to construct adenoviral vectors for cell biology and gene therapy. Most previous techniques, such as recombineering and yeast gap repair, impress more by their elegance than by their ease. In this paper, we use Cas9 ribonucleoprotein particles (RNPs) to target cleavage to specific sites in an adenoviral plasmid, then repair the break by Gibson assembly. Gibson assembly with synthetic DNA fragments has transformed basic cloning. Combining it with Cas9 RNPs, which act like highly specific restriction enzymes, makes adenoviral mutagenesis as easy as traditional plasmid cloning. We have used the approach to modify multiple sites in the adenoviral genome, but it could be applied to any large DNA virus for which the genome can be cloned in a plasmid.

## INTRODUCTION

Large DNA viruses, such as adenoviruses, contain complex genomes with multiple open reading frames, promoters, and regulatory sequences that maximize the amount of genetic information encoded in the viral genome. Understanding the life cycle of such complex viruses and converting them into viral vector systems requires precise modification of the viral genome. With genomes >35 kb, classical restriction enzyme-based cloning strategies are difficult to implement for adenoviruses because the recognition sites for standard restriction enzymes occur too often to be useful (we expect a 6 bp recognition site to occur approximately every 4^6^ bp ≈ 4 kb). An early strategy to circumvent this problem was to subdivide the adenoviral genome into segments separated by unique restriction enzymes, modify the individual segments in small plasmids, and then ligate them back together to regenerate the full-length genome (1). A recently developed modification of this approach uses a genome subdivided into five plasmids and reconstituted by DNA assembly using Gibson assembly (2, 3). If the goal is only to express a transgene from the CMV promoter in the E1 region of adenovirus 5, it is possible to use Gateway λ integrase-based cloning, for which ThermoFisher sells a destination vector (pAd/CMV/V5-DEST). This approach is highly efficient and avoids even simple cloning procedures like restriction digestion and gel purification. Most other approaches are based on using homologous recombination in bacteria, yeast, or mammalian cells to replace specific regions with modified genome fragments. Unfortunately, these approaches require specialist expertise that may not be available in standard virology labs (4–6). A widely used approach, commonly referred to as AdEasy cloning, was based on homologous recombination in bacteria between a shuttle plasmid containing the left inverted terminal repeat (ITR), the desired transgene, part of E1B, the right ITR, and a selectable marker. This shuttle plasmid was recombined in bacteria with a second plasmid encoding the missing segment of the viral genome between the E1B and E4 regions (7, 8). Cre-lox recombination, through lox-P sites inserted in the shuttle and rescue plasmids, improves the recombination efficiency, but the procedure remains complex (9). More elaborate strategies, known as recombineering, have also been used for adenoviral engineering (10–14). They require cloning of the viral genome into a bacterial artificial chromosome (BAC) for stable propagation in bacteria. Initially developed for herpesviruses, the bacterial strain expresses λ-recombinases to promote homologous recombination with incoming DNA (15, 16). Further improvements allow specific targeting of the genome through highly efficient recombination at specific sites (17). We previously described a yeast artificial chromosome (YAC)/BAC strategy that combines many of the advantages of the above systems and allows the cloning of whole genomes and modification of large genomic fragments by gap repair in yeast, as well as point mutagenesis by two-step gene replacement in yeast. This strategy, which lends itself to sequential modification at multiple sites within the viral genome, enabled us to produce replication-competent oncolytic adenoviruses with mutations in the E1, E2, E3, and E4 promoters; transfer of the packaging signal to the right end; internal deletions in E1A; green fluorescent protein (GFP) tagging of E1B 55K; insertion of RGD into Fiber; and addition of prodrug-activating enzymes, as well as making traditional vectors expressing transgenes from the E1 region (6, 18–23). Despite its versatility, this method was not widely used by other groups because it requires familiarity with yeast culture.

Clustered regularly interspaced short palindromic repeat (CRISPR) technology makes it possible to cleave double-stranded DNA at sites dictated by the sequence of the CRISPR RNA (crRNA) used to program Cas9. Because the crRNA recognizes a 20 nt target sequence, it is easy to program cleavage at unique sites in plasmids containing the full adenoviral genome. The only further sequence limitation is the requirement for a short protospacer adjacent motif (PAM) next to the crRNA target sequence. Cleavage with two guides can be used to make precise deletions, provided the ends are repaired to create valid substrates for DNA ligase. *In vitro* DNA assembly techniques like Gibson assembly have exploded in recent years to become the dominant approach used for routine plasmid mutagenesis (reviewed in Reference 24). By combining Cas9-mediated cleavage with repair by Gibson assembly, it is possible to insert or replace sequences at any site in an adenoviral plasmid. Repair with Gibson assembly also overcomes the requirement for PAMs because the sequence between the nearest available PAMs and the desired modification site can be included in the template used for Gibson assembly. Here, we show that it is possible with this combination to engineer recombinant adenoviruses in a simple overnight procedure that can be implemented in any basic laboratory at low cost. We baptized our approach AdVICE for *advanced viral genome in vitro Cas9 editing* and provided examples showing that it can be used for sequence insertion and deletion, cloning of entire adenoviral genomes from viral particles, and the construction of complex adenovirus-based vectors with enhanced coding capacity.

## MATERIALS AND METHODS

### General outline of the method

The AdVICE procedure is subdivided into four steps: (i) design and assembly of specific Cas9 ribonucleoprotein particles (RNPs) targeting the adenoviral genome at selected site(s), (ii) digestion of the plasmid harboring the adenoviral genome using Cas9 nuclease RNPs, (iii) repair with a DNA fragment containing the desired sequence by Gibson assembly, and (iv) isolation and characterization of recombinant clones. Production of recombinant viruses from selected clones then follows published procedures (13, 14).

### Design and assembly of the specific Cas9 RNP

The assembled Cas9 RNP contains the Cas9 nuclease, crRNA, and tracrRNA (or a single-guide RNA [sgRNA] if the crRNA and tracrRNA are combined in a single molecule). In the examples shown, the CRISPOR algorithm (25) was used to select crRNAs followed by PAMs that hybridize as close as possible to the desired modification site in the adenoviral genome. As a general guideline, we used a single crRNA for small insertions and point mutations. For large deletions and insertions, we used two crRNA guides to cleave near the ends of the sequence to be deleted or replaced. tracrRNA and specific crRNA were purchased from Integrated DNA Technologies (iDT, Munich, Germany). For RNP assembly, tracrRNA and crRNA were first resuspended in Tris-EDTA (TE) buffer at a final concentration of 100 µM. To anneal them, 5 µL of 100 µM crRNA and 5 µL of 100 µM tracrRNA were mixed in a 20 µL final volume of iDT duplex buffer (30 mM HEPES, pH 7.5, and 100 mM potassium acetate). Annealing was performed by heating the RNA mixture to 95°C for 5 min followed by a controlled cooling down to 25°C using a 1°C per 15 s temperature gradient. Annealed RNA (10 µL) was mixed with 4 µg of recombinant TrueCut Cas9 V2 (ThermoFisher, Illkirch, France) in a final volume of 20 µL of 1× NEB Cutsmart buffer (50 mM potassium acetate, 20 mM Tris-acetate, 10 mM magnesium acetate, and 100 µg/mL bovine serum albumin (BSA), pH 7.9; New England Biolabs, Evry, France) and allowed to assemble into RNPs for 10 min at 25°C.

### *In vitro* Cas9 digestion of adenovirus plasmid

To engineer recombinant adenoviruses using AdVICE, we began with the plasmid pAd/CMV/V5-DEST (ThermoFisher, Illkirch, France), which encodes the genome of the human adenovirus type 5 (HAdV-C5) with a Gateway attR cassette in the E1 region and a deletion in the E3 region, but any other plasmid containing the adenoviral genome should be suitable. For digestion with Cas9 RNP, 4 µg of the genome-containing plasmid in 10 µL of 1× NEB Cutsmart buffer was mixed with 20 µL of RNP and incubated at 37°C for 4 h. To stop the cleavage reaction and remove the Cas9, 1 µL proteinase K (20 mg/mL, Merck, Darmstadt, Germany) was added, and the mixture was incubated at 56°C for 10 min. The linearized DNA was then gel purified using a Nucleospin Gel and PCR Clean-up column from Macherey-Nagel (Hoerdt, France) or AMPureXP magnetic beads (Beckman Coulter, Roissy, France) and resuspended in 30 µL ultrapure water. The concentration of the purified DNA was determined spectrophotometrically with a Nanodrop (ThermoFisher).

### Preparation of repair templates and Gibson assembly

To circularize the RNP-cleaved plasmid with the desired repair templates, we used Gibson assembly with 20–30 bp overlaps at the ends of the fragments. DNA fragments containing the desired modifications (e.g., mutation, insertion, and deletion) were either PCR amplified from an existing template with primers containing the required overlaps or purchased as gBlock DNA fragments from iDT. The sequence of the primers and gBlocks is given in Table S1. To prepare viral genomic DNA from virus particles, we used the Roche High Pure Viral Nucleic Acid Extraction Kit (Roche, Basel, Switzerland). This kit includes a protease digestion step that removes all genome-associated proteins such as the covalently bound terminal protein and DNA-condensing proteins V, X, and VII. Gibson assembly was performed by mixing 200–400 ng of digested plasmid with the template at a 1:3 to 1:5 molar ratio in a final volume of 5 µL, mixed with 5 µL of 2× NEB HiFi DNA Assembly master mix (New England Biolabs), and incubated at 50°C for 15 min. Repaired DNA was precipitated with 1.5 µL NaOAcetate 3 M pH 5.2, 1 µL RNA grade glycogen (20 mg/mL) as a carrier (ThermoFisher), and 15 µL isopropanol, followed by centrifugation at 20,000 × g for 10 min at room temperature. The DNA pellet was washed with 30 µL 70% ethanol, air dried, and resuspended in 5 µL ultrapure water.

### DNA clone isolation

To recover recombinant plasmids, the Gibson assembly products were electroporated into Survival2 bacteria (ThermoFisher). Electrocompetence was achieved by growing the bacteria to an optical density of 0.8, followed by centrifugation, washing twice with ice-cold 10% glycerol, and snap freezing. For electroporation, 100 µL of electrocompetent bacteria were mixed with 5 µL of resuspended Gibson assembly product, placed in a 1 mm electroporation cuvette (Molecular BioProducts, San Diego, USA), and electroporated using a GenePulser Xcell (Biorad, Hercules, USA) at 1,800 V, 25 µF, and 200 Ω. Immediately after electroporation, the bacteria were resuspended in 1 mL of Luria Bertani (LB) medium and incubated for 30–60 min at 37°C to express the bacterial resistance genes. About 100 µL was subsequently plated on an LB agar plate supplemented with ampicillin at 100 µg/mL, plus chloramphenicol 35 µg/mL if the Gateway cassette was present, and incubated overnight at 37°C. Individual colonies were picked and processed using standard protocols for plasmid purification. The purified plasmids were analyzed for the presence of the desired modification using restriction enzyme digest or PCR and validated by Sanger and Nanopore whole-plasmid sequencing.

### Packaging of virus

Plasmids containing the viral genome were cleaved with *Pac*I to release the viral DNA, and then transfected into packaging cells following standard protocols (14). For the production of E4-deleted vectors, E1 complementing HEK cells stably overexpressing αvβ5 integrins (26) were infected with a lentiviral vector (pTTBC28) expressing E4 orf6/7 from a tetracycline-inducible promoter and selected with puromycin to give 293N4 cells. To produce pTTBC28, a fragment of Ad5 DNA containing E4 orf6 and orf6/7 was amplified with attB-tailed oligos (GGGACAAGTTTGTACAAAAAAGCAGGCTTCACCatgactacgtccggcgttc and GGGGACCACTTTGTACAAGAAAGCTGGGTtcacagaaccctagtattc), inserted in pDONR221, sequenced, and transferred to pCW57.1 (Addgene 41393) by Gateway cloning. E4 orf6/7 expression was induced in 293N4 cells with 1 µg/mL doxycycline for 12 h before transfection of DNA or infection with virus. E4-deleted virus was expanded through three cycles of infection in 293N4 cells, then double-banded by ultracentrifugation in CsCl gradients and dialyzed into phosphate-buffered saline (PBS)/10% glycerol buffer. Recombinant adenoviruses were produced under authorizations #6745 and #11968 from the French Ministry of Higher Education and Research.

### Immunofluorescence and western blotting

For the fluorescence microscopy, cells were grown on coverslips and infected as indicated. To process the cells, coverslips were fixed in 4% paraformaldehyde in PBS and stained with primary and secondary antibodies diluted in IF buffer (10% fetal calf serum, 0.1% saponin in PBS) and incubated at 37°C for 1 h each, followed by mounting in DAPI-containing DAKO mounting medium (Agilent, Santa Clara, USA) for nuclear counterstaining. Fluorescent microscopy images were taken on a Leica DMi-6000 inverted microscope equipped with an Aura III light source from Lumencor and a Hamamatsu Orca Flash 4 Camera and a Cicero spinning disk setting piloted MetaMorph and mounted in ImageJ software. Inducible E4 orf6 expression was confirmed by treating 293N4 cells with 1 µg/mL doxycycline for 24 h then western blotting with rabbit polyclonal antibodies against the N and C-termini of E4 orf6 diluted 1:10,000 (gift from Dr. Branton) (27). Cas9 expression was verified by western blotting with monoclonal antibody clone 7A9-3A3 (CST#14697; Cell Signaling Technology, Danvers, USA) diluted 1/1,000. Adenoviral proteins were detected using a rabbit serum raised against purified Ad5 particles (gift from Dr. Russell). Protein V and DNA binding protein (DBP) were detected using rabbit serum (gift from Dr. Nagata) and a mouse monoclonal antibody (gift from Dr. Berk), respectively. Primary antibodies were detected with horseradish peroxidase (HRP)-conjugated goat secondary antibodies from Cytiva (Amersham, UK) diluted 1:2,500 and visualized by chemiluminescence with a Chemidoc (BioRad) or using secondary Alexa-594 conjugated donkey-anti mouse or rabbit antibodies diluted at 1:300 (Thermo Fisher).

## RESULTS

To demonstrate the versatility of the AdVICE technique, we created adenoviruses that cover a large area of fundamental and applied adenovirus research applications. This includes recreating some vectors made previously by recombineering. We explored the creative potential of AdVICE by showing that it can be used for the *de novo*, multi-step construction of an adenovirus vector suitable for delivering very large transgenes for gene editing. As outlined below, the main advantage is the speed, simplicity, versatility, and high efficiency with which adenovirus genome modifications can be achieved.

### Sequence insertion (single cut)

**Fig 1:**
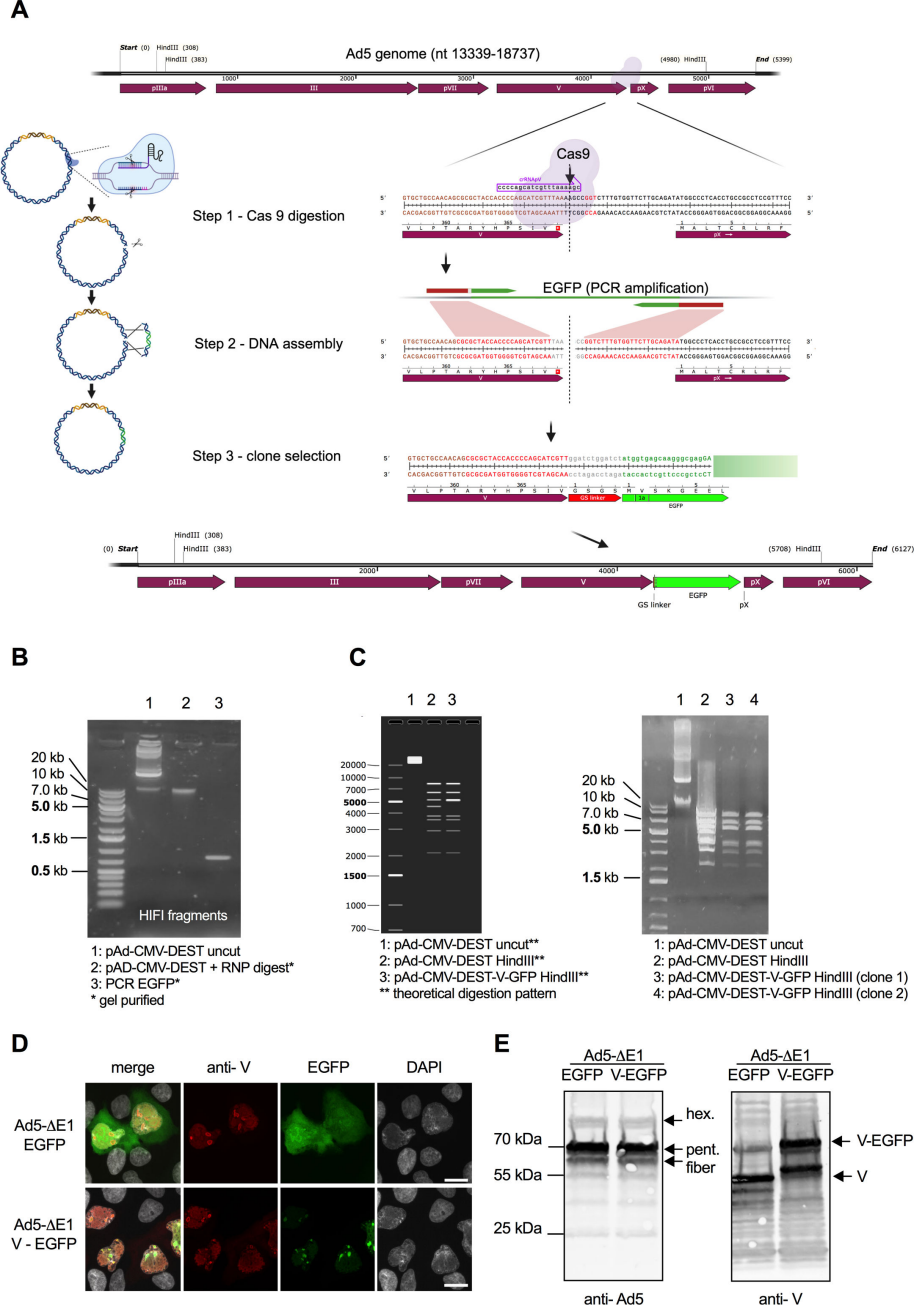
Fusion of protein V to EGFP. (**A**) Workflow for the fusion of EGFP to the C-terminus of protein V. (**B**) Agarose gel showing the parental plasmid before and after RNP digestion and the EGFP repair template after PCR amplification. Please note that any potential line artifacts in the background of gel images may be attributable to the scanning process. (**C**) Expected (left) and observed (right) pattern of bands following *Hin*dIII digestion of the parental and protein V-EGFP viruses. (**D**) Immunofluorescence images of 293 cells infected with Ad5 expressing EGFP from the CMV promoter in the E1 locus and Ad5 expressing EGFP-tagged protein V. Cells were fixed, probed with anti-protein V antibody, and stained with DAPI. Scale bar, 10 µm. Green, EGFP; red, protein V; gray, DAPI. (**E**) Western blot of Ad5 expressing EGFP from the CMV promoter in the E1 locus and Ad5 expressing EGFP-tagged protein V, probed with antibody against Ad5 virion proteins and an antibody against protein V. Protein V and EGFP-tagged Protein V are shown with arrows.

In our first example, we fused the C-terminus of adenovirus protein V to enhanced green fluorescent protein (EGFP) (Fig. 1). GFP tagging was previously used to follow the fate of protein V during adenovirus infection (28) and to identify late virion accumulation compartments in adenovirus-infected cells (29) using virus engineered by either transposon mutagenesis (30) or homologous recombination in a *ccdB* counterselection system (31). To recreate the protein V-EGFP fusion protein with the AdVICE technique, we started from the plasmid pAd/CMV/V5-DEST. We used the CRISPOR program to find potential crRNA sequences as close as possible to the stop codon of protein V (Fig. 1A; Table S1) and selected a crRNA guide expected to program a dsDNA break four nucleotides downstream of the terminal valine codon in the protein V coding sequence. Assembly of the Cas9 RNP was performed as described in Materials and Methods. To cleave the plasmid, 4 µg of pAd/CMV/V5-DEST was incubated with RNP at 37°C for 4 h followed by proteinase K digestion. Two microliters of the mixture was retained for analysis, and the rest of the linearized DNA was purified using AMPureXP beads and resuspended in 30 µL ultrapure water. To generate the template containing EGFP fused to the C-terminus of protein V, we designed PCR primers (Table S1) that add a Gly-Ser-Gly-Ser linker between protein V and EGFP and include 25 bp tails that overlap with the ends of the viral DNA at the cleavage site. The resulting PCR product was gel-purified on a 1% agarose gel (Fig. 1B), and Gibson assembly was performed with the NEB HiFi DNA Assembly Kit using 400 ng of the linearized vector and 25 ng of the PCR product. The mixture was incubated at 50°C for 15 min then the DNA was precipitated, resuspended in 5 µL ultrapure water, electroporated into Survival2 electrocompetent bacteria, and plated on LB agar plates supplemented with ampicillin + chloramphenicol, yielding >100 colonies. Colonies were tested by PCR for the presence of the EGFP sequence. Plasmid preps from positive clones were *Hin*dIII digested (Fig. 1C) to confirm EGFP insertion at the C-terminus of protein V and sequenced to rule out PCR mutations. Two validated clones were converted to viruses using standard methods, and the resulting virus was shown to express EGFP-tagged protein V (Fig. 1D and E), in line with previous publications (28, 29).

### Sequence replacement (double cut)

**Fig 2:**
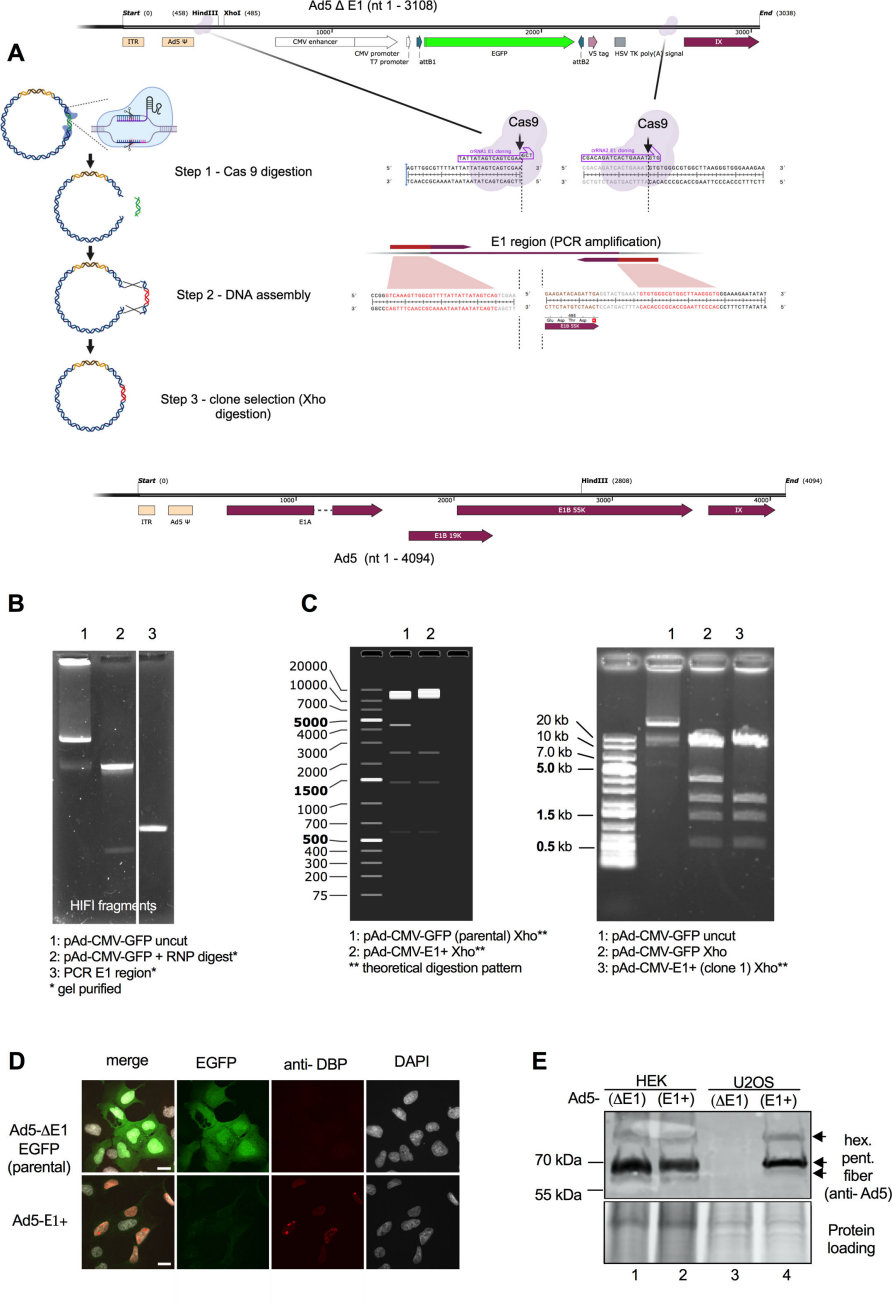
Reversion of the E1 region in a first-generation adenoviral expression vector to the wild-type E1 sequence. (**A**) Workflow for replacement of the CMV expression cassette by the wild-type E1 region. (**B**) Agarose gel showing the parental plasmid before and after RNP digestion and the E1 repair template after PCR amplification. (**C**) Expected (left) and observed (right) pattern of bands following *Xho*I digestion of the parental and E1-reverted viruses. (**D**) Fluorescence images of U2OS cells infected with the parental vector expressing EGFP from the CMV promoter in the E1 locus and with the same virus after reversion of the E1 region to wild type. Fixed cells were probed with anti-DBP antibody at 24 h post-infection to show replication compartments. Scale bar, 10 µm. Green, EGFP; red, DBP; gray, DAPI. (**E**) Western blot for expression of adenoviral proteins after infection of E1-complementing HEK 293T and non-complementing U2OS cells with parental E1-deficient virus and with the same virus after reversion of the E1 region to wild type.

In our second example, we converted a first-generation adenoviral expression vector back into a replication-competent adenovirus by replacing the transgene expression cassette with the original E1 sequence (Fig. 2). We started with pAd/CMV/V5-DEST into which GFP had been inserted by Gateway cloning. We designed crRNAs that target sites in the viral genome flanking the transgene expression cassette. For the upstream crRNA, we targeted a site immediately following the adenoviral packaging signal, and for the downstream crRNA, we targeted a site between the E1B55K stop codon and the protein IX start codon (Table S1; Fig. 2A). RNPs were assembled, and the plasmid was cleaved as described above. Successful cleavage was demonstrated by the release of the 2,135 bp fragment containing the EGFP expression cassette (Fig. 2B). The cleaved plasmid was purified with AMPureXP beads. In parallel, we PCR-amplified and gel-purified the E1 region from a wild-type virus (HAdV-C5 from ATCC) with primers giving 20 bp overlaps with the cleaved vector (Table S1; Fig. 2B). We performed Gibson assembly with 300 ng of linearized plasmid and 90 ng of the E1 PCR product (1:3 molar ratio) at 50°C for 15 min, followed by precipitation with isopropanol and electroporation of the recombined DNA into Survival2 bacteria. Plasmid DNA was purified from randomly picked colonies and digested with *Xho*I to detect loss of the *Xho*I site in the transgene expression cassette (Fig. 2C). Successful reintroduction of the E1 region was then verified by sequencing. Correctly edited plasmid was digested with *Pac*I and converted into a virus by transfection of HEK 293 cells. Loss of EGFP expression and gain of replication competence were confirmed by infecting U2OS cells with the E1-region reconstituted virus (Fig. 2D and E).

### Genome trapping from biological material

**Fig 3:**
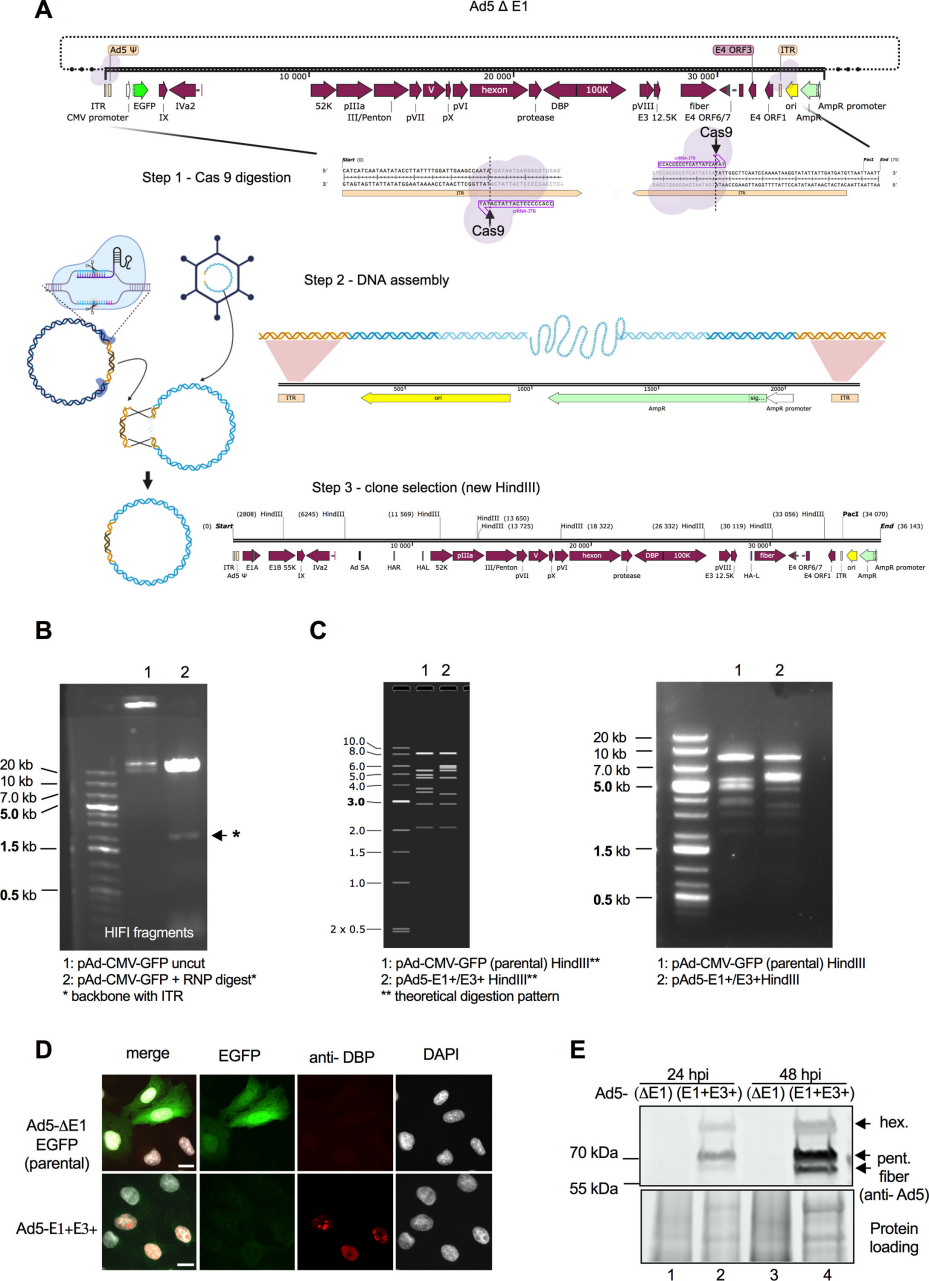
Genome trapping from adenoviral virions using AdVICE. (**A**) Workflow for genome trapping and circularization. (**B**) Agarose gel showing the parental plasmid before and after RNP digestion. The plasmid fragment containing the ITRs is shown by a black arrow. (**C**) Expected (left) and observed (right) pattern of bands following *Hin*dIII digestion of the parental and genome-trapped plasmids. For panels B and C, any potential line artifacts in the background of gel images may be attributable to the scanning process. (**D**) Fluorescence images of U2OS cells infected with the parental vector expressing EGFP from the CMV promoter in the E1 locus and with the replication-competent full Ad5 virus after genome trapping. Fixed cells were probed with anti-DBP antibody at 24 h post-infection (hpi) to show replication compartments. Scale bar, 10 µm. Green, EGFP; red, DBP; gray, DAPI. (**E**) Western blot for adenoviral proteins after infection of U2OS cells with parental non-replicative vector and with the replication-competent full Ad5 virus after genome trapping, 24 and 48 h post-infection.

We next investigated if the AdVICE approach could be used to clone entire genomes from intact virus. As proof of concept, we extracted DNA from viral particles and attempted to repair a vector by Gibson assembly through overlaps between the ITRs in the vector and the ITRs in the DNA purified from the viral particles. To prepare the vector backbone, we used CRISPOR to design a single crRNA targeting sites in the ITRs located 40 bp from the ends of the adenoviral genome (Table S1; Fig. 3). Cutting the plasmid with Cas9 RNPs containing this crRNA should release the entire pAd/CMV/V5-DEST genome while retaining the plasmid backbone with 40 bp of ITR at the ends of the linearized vector. Cas9 RNPs were assembled, and cleavage of pAd5-CMV-GFP plasmid was performed as described above (Fig. 3B). Gibson assembly with 10 ng of linearized vector (corresponding to 175 ng of input plasmid) and 500 ng of DNA purified from viral particles was used to clone the viral genome into the plasmid. The assembled DNA was precipitated with isopropanol and electroporated into bacteria. Plasmid DNA extracted from randomly selected clones was digested with *Hin*dIII to reveal the typical digestion pattern of entire adenoviral genomes (Fig. 3C). To verify the “trapping” of functional viral genomes, one plasmid was digested with *Pac*1 and transfected into HEK 293 cells. The resulting virus was tested for replication competence and the absence of GFP expression on non-complementing U2OS cells (Fig. 3D and E).

### Vector construction: adenovirus vectors expressing large transgenes

**Fig 4:**
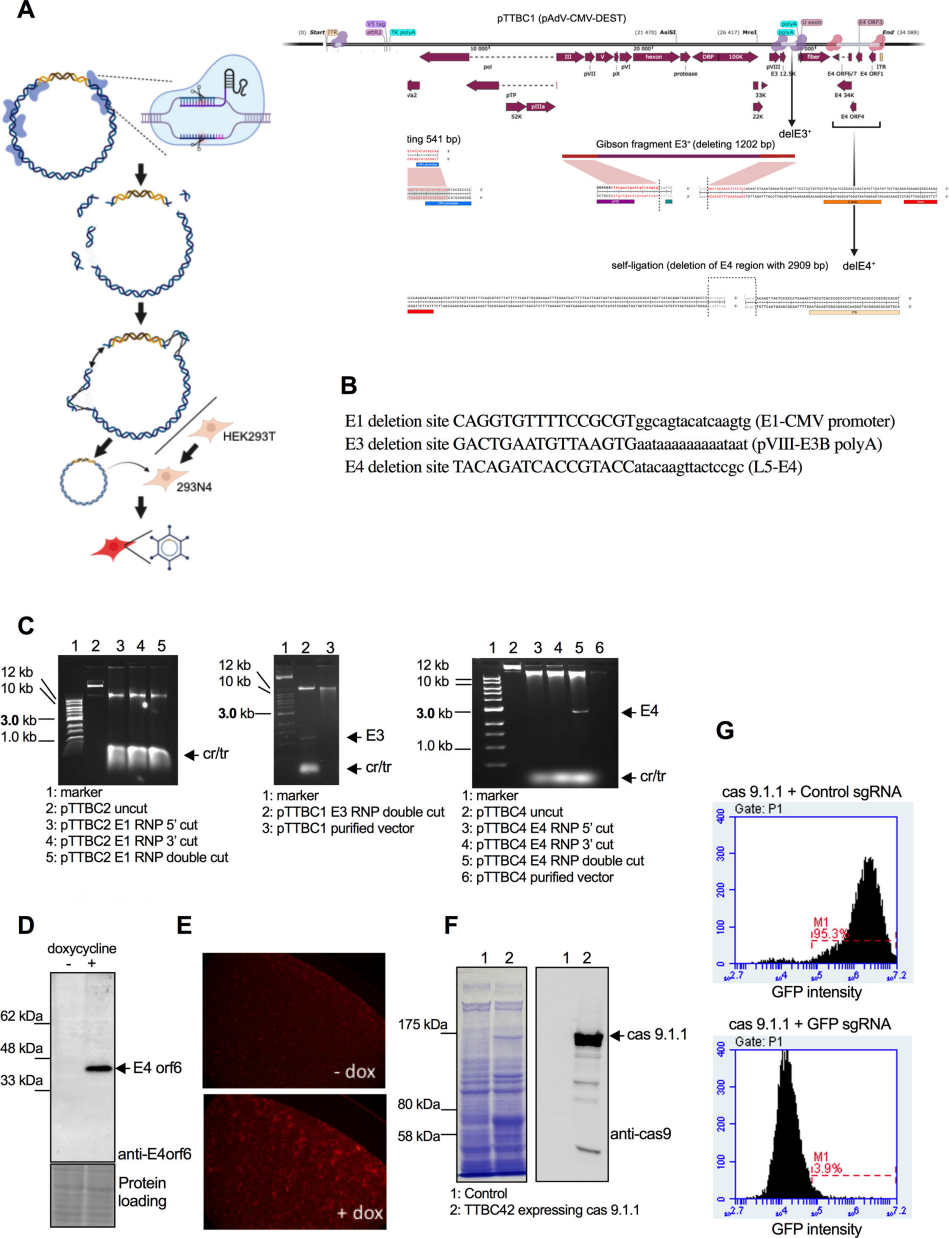
Construction of a Cas9.1.1 expression vector with the AdVICE technique. (**A**) Schematic diagram showing the genome organization of the viruses with E1, E3, and E4 deletions. (**B**) The sequence at the E1, E3, and E4 deletion breakpoints is shown. (**C**) Gels showing cleavage of the parental vectors to create the deletions in E1, E3, and E4. A fragment released by the double digests is visible for E3 (1,208 bp) and E4 (2,909 bp) but was not resolved for E1 (541 bp). cr/tr, CRISPR, and tracr RNAs. The gel-purified vectors used for Gibson Assembly and self-ligation are shown for the E3 and E4 digests. (**D**) Western blot showing E4 orf6 34K protein expression 1 day after treatment of 293N4 cells with 1 µg/mL doxycycline. (**E**) tdTomato fluorescence showing that 293N4 cells are more permissive for the production of E4-deleted vectors after induction of E4 orf6/7 expression by treatment with doxycycline. 293N4 cells shown 11 days after transfection of pTTBC49 DNA cleaved with *Pac*I. Red, tdTomato. (**F**) Coomassie gel and western blot showing the expression of Cas9.1.1 after infection of MDA-MB-231 cells with vTTBC42 (Cas9.1.1 in the ΔE13 virus). (**G**) Histograms showing flow cytometry for the number of green cells after infection of MDA-MB-231 cells with vTTBC42. The cells contain an integrated lentiviral vector expressing GFP and have been superinfected with lentiviral vectors expressing either control sgRNA or sgRNA targeting GFP. Infection with the Cas9.1.1 adenoviral vector has almost completely eliminated GFP fluorescence (3.9% vs 95% green cells).

In the age of gene editing, viral vectors are often used to deliver Cas9 variants and fusion proteins. Many of these inserts are too large for lentiviral expression, resulting in unacceptably low lentiviral titers. Gutless adenoviral vectors (32) accept large inserts, but they are only mastered by a few expert labs. In contrast, E1-deleted vectors can be produced by any lab with basic viral vector expertise. Classic helper-independent adenoviral vectors like pAd/CMV/V5-DEST contain deletions in the E1 and E3 regions which date from before the invention of techniques that allow precise selection of the deletion boundaries (for example, dl309, in which 745 bp of E3 DNA is replaced with 642 bp of salmon DNA; [33]). To increase the capacity of pAd/CMV/V5-DEST, we used AdVICE to precisely engineer a larger deletion in E1 that removes an additional 541 bp while preserving all of the packaging signals. In E3, we deleted an additional 1,202 bp while retaining the E3B polyA signal for pVIII expression and preserving the U exon. In E4, we removed all of the orfs with a 2,909 bp deletion. The vectors and intermediates produced with the AdVICE technique are listed in Table 1, with additional details in Table S1. Figure 4 shows an overview of the approach (Fig. 4A), including the sequence at the E1, E3, and E4 deletion breakpoints (Fig. 4B). The deletions were made by cleaving the parental plasmid with Cas9 RNPs flanking the regions to be deleted (the crRNA spacers are listed in Table S1). Figure 4C shows the respective RNP-cleaved vectors. Gibson Assembly was performed in the E1 and E3 regions (Fig. 4A) with templates containing the desired final sequences (the sequence of the inserts is shown in Table S1). The E4 deletion was made by self-ligation without a template. E4 orf6/7 is the only E4 orf required for virus production in 293 cells. To propagate E4-deleted viruses, we made a packaging cell line (293N4) that expresses E4 orf6/7 from an inducible promoter (Fig. 4D). As shown previously, this allows the production of E4-deleted viruses (7, 34). Since E4-deleted viruses do not make well-demarcated plaques, we inserted tdTomato into our vector as a control to verify that the 293N4 cells can produce E4-deleted viruses (Fig. 4E). To demonstrate that our new vectors with improved cloning capacity can be used for gene editing, we used the new E1/E3-deleted vector to express Cas9 and the E1/E3/E4-deleted vector to express a prime editor. For the former, we cloned a high-fidelity Cas9 mutant (Cas9.1.1; [35]) by Gateway cloning into the attR sites in E1 (creating pTTBC42) and showed that it gives high-level expression of Cas9. There was a prominent band of Cas9 detectable by Coomassie staining and verified by western blotting (Fig. 4F). When we infected a cell line stably expressing GFP with a virus produced from the pTTBC42 vector, we achieved full suppression of GFP expression in cells containing an sgRNA targeting GFP, demonstrating full functionality of our new adenoviral Cas9 expression vector (Fig. 4G).

**TABLE 1:** Gene editing and control vectors, showing the inserts, deletions, and genome size

Name	Ad5 backbone	Genome size	Genome (%)	Insert
Control vectors				
pTTBC1/Ad-CMV-dest	(old ΔE13)	34,605	96.3	Empty vector
pTTBC 2	(old ΔE1) ΔE3	33,513	93.3	Empty vector
pTTBC 3	ΔE13	32,972	91.7	Empty vector
pTTBC 4	ΔE134	30,431	84.7	Empty vector
pTTBC 6	ΔE134 + orf6	31,300	87.1	Empty vector
pTTBC 48	ΔE13	32,253	89.7	tdTomato
pTTBC 49	ΔE134	29,344	81.7	tdTomato
Gene editing vectors				
pTTBC 30	ΔE13	34,987	97.4	Cas9wt
pTTBC 42	ΔE13	34,987	97.4	Cas9.1.1
pTTBC 14	ΔE134	36,605	101.9	PEmax-dnMLH1
pTTBC 62	ΔE134 + orf6	37,842	105.3	PEmax-dnMLH1
pTTBC 67	ΔE134	37,059	103.1	PEmax-dnMLH1 PEG-AR 10 bp
pTTBC 68	ΔE134	37,056	103.1	PEmax-dnMLH1 PEG-AR 7 bp

**Fig 5:**
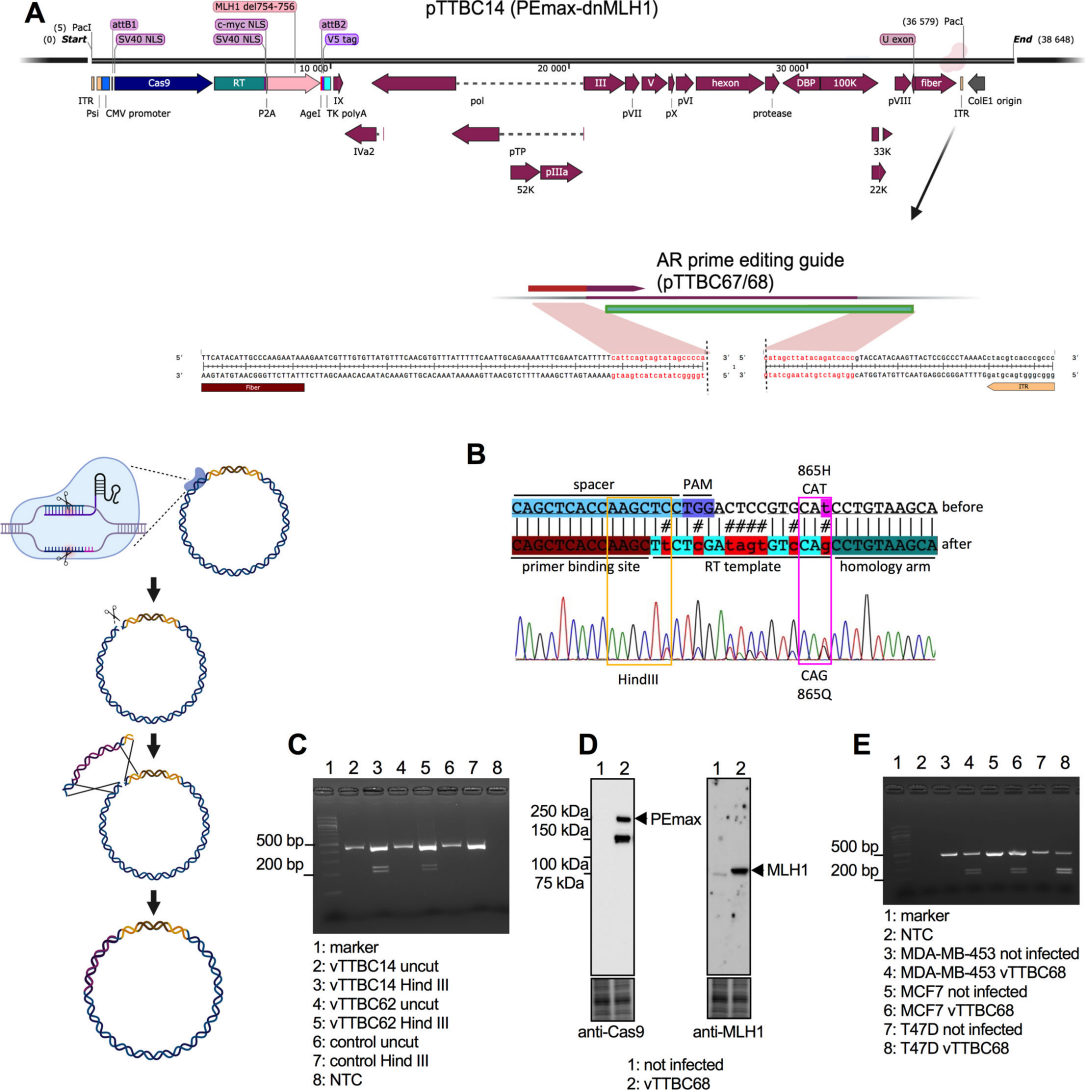
Construction of a prime editing vector with the AdVICE technique. (**A**) Schematic diagram showing insertion of the PEG targeting *AR* at the junction between L5 and E4. (**B**) Design of the AR PEG and outcome of editing. The upper row shows the *AR* sequence around codon 856 in the MDA-MB-453 cell line. The crRNA (spacer) directs nicking by Cas9 17 nt upstream of the mutation. The lower row shows the genomic sequence after editing. The primer binding site in the PEG RNA forms base pairs with the displaced genomic strand before the nick. The RT in the prime editor then copies the template starting at the nick. To prevent renicking, the template contains silent mutations that preserve the AR protein sequence. One of these mutations creates a *Hin*dIII site. The G in codon 865 that is mutated to T to revert the codon to glutamine is immediately before the homology arm. Below the alignment, the Sanger sequence of chromosomal DNA edited with pTTBC14 is shown. (**C**) Gel showing *Hin*dIII cleavage of a PCR fragment amplified from MDA-MB-453 genomic DNA after editing. vTTBC14 and 62 both contain the prime editor; the control virus expresses a form of catalytically inactive Cas9. All of the cells contain the PEG expressed from a lentiviral vector, but editing is only seen in the presence of the prime editor. (**D**) Western blot showing expression of PEmax and MLH1 from vTTBC68. Note that PEmax readily undergoes cleavage at the junction between Cas9 and RT, leading to the formation of a lower band that runs at the size of Cas9. (**E**) Gel showing *Hin*dIII cleavage of a PCR fragment amplified from MDA-MB-453, MCF7, and T47D genomic DNA after editing with vTTBC68.

The newly created vector with optimized E1/E3/E4-deletions has an even higher cloning capacity as it retains only 84.7% of the full genome length compared to 96.3% in the parental vector (Table S1). To show that the additional deletions permit the cloning and expression of very large inserts, we chose to express one of the largest transgenes currently used for gene editing: the PEmax-2A-dnMLH1 cassette developed by the Liu lab (36). We inserted it by Gateway cloning into the attR sites in the E1 region of pTTBC4, creating pTTBC14 (Fig. 5A). Our ultimate goal is to revert the *AR* codon 865H mutation in MDA-MB-453 cells to wild type (865Q) by prime editing. The *AR* Q865H mutation in MDA-MB-453 cells is thought to attenuate the response to androgen (37), potentially explaining the pathogenesis of the molecular apocrine breast cancer subtype we previously described (38). The prime editing guide (PEG) RNA is aligned to the mutant genomic *AR* sequence in Fig. 5B. In addition to reverting the mutation, successful editing introduces a *Hin*dIII site. We first tested editing by the adenoviral vector when the PEG RNA was supplied on a lentiviral vector. This showed that the adenoviral prime editor was indeed able to edit the *AR* gene (Fig. 5C). A PCR fragment containing codon 865 acquired the expected *Hin*dIII site, and Sanger sequencing confirmed successful editing. In addition to the ΔE134 vector (vTTBC14), Fig. 5C shows editing by a helper-independent vector (vTTBC62) made with the AdVICE technique that retains E4orf6/7, but its genome is 105.3% of wild-type Ad5, so we did not pursue this approach. Since lentiviral vectors permanently modify the cells, we next used the AdVICE technique to insert the *AR* PEG into the adenoviral vector itself to allow transient expression of both the prime editor and the PEG. To do this, we cleaved the pTTBC14 plasmid with a single RNP targeting the L5–E4 junction and performed Gibson Assembly with a DNA fragment containing the *AR* PEG, to give pTTBC68, as shown in Fig. 5A. The genome size of vTTBC68 is 103.1% of wild-type Ad5 (Table S1), which is well below the 105% limit for viral genome stability (39). After reverse transcription of the PEG template by the prime editor, the homology region for flap exchange is 7 bp in vTTBC68. Figure 5D shows by western blotting for Cas9 and MLH1 that both transgenes are expressed by cells infected with vTTBC68. The spacer in the *AR* PEG is not specific to the *AR* Q865H mutation in MDA-MB-453, so the PEG can be used to insert the *Hin*dIII site into cells with wild-type *AR*. Figure 5E confirms that the prime editor and PEG expressed by vTTBC68 can edit the *AR* locus in MDA-MB-453, MCF7, and T47D cells: editing introduces the *Hin*dIII shown in Fig. 5B, leading to the formation of the lower bands seen after digestion of the PCR products with *Hin*dIII in Fig. 5E.

## DISCUSSION

We have described a simple strategy to engineer new adenoviruses and adenoviral vectors by combining CRISPR technology with Gibson assembly to cleave adenoviral plasmids at specific sites and repair them with linear DNA fragments containing the desired sequence. Many techniques for adenoviral engineering have been described, but to our knowledge, this is by far the simplest and quickest because it does not require complicated vectors and can be completed in 24 h. We have shown that it can be used for adenoviruses, but the technique could be used for any large DNA virus whose genome can be cloned in a plasmid, for example, herpesviruses. Adenoviruses and herpesviruses have been modified with recombination-based strategies that are commonly referred to as *recombineering*. Recombineering requires cloning of the viral genome into a BAC for stable propagation in bacteria. The bacterial strain expresses λ-recombinases to promote homologous recombination with incoming DNA (15, 16). The most widely used strategy to modify viral genomes is based on a two-step procedure that first integrates a galactokinase-kanamycin (*galK*) cassette by positive selection, then replaces it with the desired sequence by negative selection (17, 40). Our approach would greatly simplify this procedure. The main advantages of AdVICE are that it can be performed in a single day, it does not require complicated vectors or recombinase-expressing hosts, and the success rate compares to recombineering. In terms of speed, AdenoBuilder might be the most comparable approach to AdVICE. This approach uses an adenoviral genome subdivided into five parts and reassembled by Gibson assembly after conventional manipulation of the subfragments (2) (3). Despite their elegance, most other previously described procedures for the construction of adenoviral vectors, such as recombineering or two-step gene replacement in yeast, require up to 2 weeks in an experienced lab (6). Cleavage with Cas9 leaves DNA breaks that require processing with T4 DNA polymerase if no template is supplied, but this gives a mixture of deletions (41). Repair with T4 DNA polymerase is unnecessary if the break is repaired by Gibson assembly from a template and has the added advantage that essentially all of the recombinants contain the desired sequence. This contrasts with the outcome of homology-directed genome editing with Cas9 in mammalian cells, where a mixture of deletions and templated insertions is commonly seen because cleavage by NLS-tagged Cas9 typically starts before the DNA template reaches the nucleus.

We have shown that the technique can be used to create specific mutants for studies on adenovirus biology. In addition to making deletions, insertions, and point mutations, the technique can be used to clone entire genomes from virions. This is analogous to cloning entire genomes by gap repair in yeast, a technique we previously used to clone AdC clinical isolates in yeast (42). Gap repair in yeast is extremely efficient, but the YAC/BAC must subsequently be transferred to bacteria for the purification of sufficient DNA for transfection into 293 cells. The YAC/BAC approach requires familiarity with yeast culture, which blunted enthusiasm for the technique.

Beyond studies on adenovirology, a large community of scientists uses adenoviral vectors for gene expression and gene therapy. A classic limitation in these studies is the 105% packaging limit, relative to the length of the viral genome, which is dictated by the fixed volume inside the icosahedral capsid (39) (43). Optimizing vectors by deleting unwanted viral sequences was rarely done in the past because of the difficulty of making precise deletions. The AdVICE technique allowed us to optimize the deletions in E1, E3, and E4 to create space for one of the largest inserts currently used for gene editing. Production of E4-deleted vectors requires complementation by expression of E4 orf6/7 in the packaging cells. Our 293N4 cell line facilitates the conversion of adenoviral plasmids into virus because E4 orf6/7 dimerizes E2F on the E2 promoter (44), leading to increased expression of viral replication proteins immediately after transfection, and the cells over-express αvβ5 integrins (26), leading to increased uptake of released virus by surrounding cells. It is not the subject of this report, but these effects are not restricted to E4-deleted viruses, so the 293N4 cell line could potentially be used to promote the conversion of any adenoviral plasmids to virus, a step that can be troublesome with highly engineered viruses.

Adenoviral vectors commonly lead to transient high-level transgene expression. This is a disadvantage in studies requiring normal levels of gene expression, but for gene editing, it is exactly what is required. We based our expression vectors on an existing Gateway plasmid that greatly simplifies the insertion of transgenes. We show that this plasmid, with our optimized E1 and E3 deletions, allows high-level expression of a high-fidelity mutant of Cas9. This vector lends itself to testing potentially interesting mutants of Cas9, for example, a conditionally destabilized version of Cas9 (45). To demonstrate the utility of the AdVICE approach, we further deleted the E4 region to produce a vector with a theoretical cloning capacity of 9.9 kb, based on a limit of 105% of the Ad5 genome length (39). We used this vector to express a prime editor, PEmax, together with a dominant negative mutant of MLH1 to prevent removal of the edited sequence (46). Most of the exogenous protein runs at the molecular weight of Cas9 alone, suggesting that the Cas9-reverse transcriptase fusion protein breaks at the junction between the two proteins in the cell or during lysis for western blotting. Several groups, including that of David Liu, have now reported that RT does not need to be fused to Cas9 for editing to occur (reviewed in Reference 47), so cleavage at the linker within the cell should not prevent editing. We introduced silent mutations between the *AR* Q865H mutation and the cleavage site to prevent flap exchange before the Q865H site. This means that there were eight mismatches in the 17 nt template. We have not performed an extensive mutagenesis screen to compare different spacers, primer binding sites, or homology regions. The PREDICT server (48) recommended shortening the homology region, so we tested 7 bp and 10 bp homology regions but saw similar editing results. We suspect that editing does not go beyond 50% because one mutant allele is heterochromatinized (*AR* is on the X chromosome) (49).

We conclude that the AdVICE technique greatly simplifies the construction of adenoviral vectors, consistent with reports from other groups (50, 51). The ability to engineer new adenoviruses and adenoviral vectors in a single-day procedure should lead to more widespread use of adenoviral vectors, which have hitherto been restricted to specialist labs because of the daunting complexity of the cloning procedures required to make the vectors.

## Data Availability

All data and plasmids will be made fully available upon reasonable request.

## SUPPLEMENTAL MATERIAL

Table S1 jvi.02265-24-s0001.xlsx Parental vectors, guide RNAs, inserts, and construction technique used for this study.

ASM does not own the copyrights to Supplemental Material that may be linked to, or accessed through, an article. The authors have granted ASM a non-exclusive, world-wide license to publish the Supplemental Material files. Please contact the corresponding author directly for reuse.
